# Thereaputical Actions and Uses of Turpentine

**Published:** 1871-10

**Authors:** 


### THERAPEUTIC ACTIONS AND USES OF TURPENTINE.

Dr. Warburton Begbie read a paper on this subject before the Medico-Chirurgical Society of Edinburgh. He gave a brief sketch of the ancient history of the drug from the time of Hippocrates, with a notice of the various forms in which the oleoresins of the conferæ are used or have been used in therapeutics. Oil of turpentine was described as being irritant and stimulant, quickening the circulation and augmenting the temperature of the body. In larger doses it produces a sort of intoxication; in drachm doses it is hynoptic. Externally it is a valuable rubefacient, and is absorbed by the skin so as very soon to be recognized in the breath, and by its characteristic violaceous odor in the urine. The production of this violaceous odor in its perfection seems to be a test of the integrity of the urinary organs, as it is less marked in disease of the kidneys. The therapeutic actions and uses of turpentine are various. 1. As a cathartic it is uncertain, but along with castor oil it is useful in cases of obstinate obstruction and tympanitis. 2. As an anthelmintic it is chiefly used as a cure for tapeworm; also, in the form of enema, it destroys ascarides and lumbrici. 3. Though turpentine sometimes causes hæmaturia, it cures passive hemorrhages. It is useful in purpura, probably acting through the nervous spstem; and it is also useful in hæmoptysis, hæmaturia, and uterine hemorrhages. 4. As a stimulant, it is especially valuable in adynamic fevers; as in the stupor of typhus, in certain kinds of delirium, and in the latter stages of enteric fever with a dry tongue. 5 In certain nervous diseases, such as epilepsy and chorea, it is said to be very useful; but in epilepsy it is supplanted by bromide of potassium, and in chorea by arsenic. In certain forms of sciatica and crural or brachial neuralgia in the aged, twenty-minim doses thrice daily have a very good effect. In the nervous headaches of delicate females, and the headache which is induced by fatigue, it is a better stimulant even than strong tea, and without the effect which tea so often has of banishing sleep. 6. In all chronic discharges from mucous membranes, such as chronic and fetid bronchitis, it is very useful, and even is advantageous in gangrene of the lung in checking the fetor. Under this head some interesting cases were given of gangrene of lung depending on the presence of foreign bodies.—*Brit. Med. Journal.*

